# Supplementary material for: Mid-Holocene extreme precipitation in the Tibesti, Central Sahara
Source: Nat Commun. 2025 Aug 11;16:7426. doi: 10.1038/s41467-025-62769-9 (PMC12340050; doi:10.1038/s41467-025-62769-9)
Supplement: Supplementary file 1 — Supplementary Information [file 41467_2025_62769_MOESM1_ESM.pdf]

Mid-Holocene extreme precipitation in the Tibesti, Central Sahara

Supplementary information

Philipp Hoelzmann[1]*, Martin Claussen[2,3]*, Anne Dallmeyer[2], Frank Darius[1], Michèle Dinies[1], Christian Reinhardt-Imjela[1], Leonore Jungandreas[4], Birgit Schröder[5], Stefan Kröpelin[6]

*These authors contributed equally to this work: Philipp Hoelzmann and Martin Claussen

Affiliations:

[1] Institute of Geographical Sciences, Freie Universität Berlin, Malteser Str. 74-100, 12246 Berlin, Germany.
[2] Max-Planck-Institut für Meteorologie, Bundesstr. 53, 20146 Hamburg, Germany.
[3] Center for Earth System Research and Sustainability (CEN), Universität Hamburg, Bundesstr. 53, 20146 Hamburg, Germany.
[4] German Centre for Integrative Biodiversity Research (iDiv), Leipzig, Germany, now at: Institute for Earth System Science and Remote Sensing, Universität Leipzig, Leipzig, Germany.
[5] GFZ Helmholtz Centre for Geosciences, Telegrafenberg, 14473 Potsdam, Germany.
[6] Institute of Prehistoric Archaeology - African Archaeology, Universität zu Köln, Jennerstr. 8, 50823 Köln, Germany.

*Correspondence to philipp.hoelzmann@fu-berlin.de or martin.claussen@mpimet.mpg.de

This supplementary information contains

Supplementary Notes

- General geologic setting
- Lithology
- Dating and age depth model
- Palaeolake evolution
- Assessment of the climate modelling results

Supplementary Figures

- Supplementary Fig. 1. Schematic profile from the Pic Toussidé into the Trou au Natron.
- Supplementary Fig. 2. Schematic profile from the Emi Koussi summit caldera with the Era Kohor crater.
- Supplementary Fig. 3. Age depth model of the Trou au Natron palaeolake section JK48.
- Supplementary Fig. 4. Age depth model of the Trou au Natron palaeolake section W99.
- Supplementary Fig. 5. Age depth model of the Era Kohor palaeolake section W566.
- Supplementary Fig. 6. Timeseries of monthly mean precipitation rates averaged over the Tibesti Mountain in the 40 km simulation.
- Supplementary Fig. 7. Evaluation of the climate model simulation with respect to precipitation.

Supplementary Tables

- Supplementary Table 1. Radiocarbon datings.
- Supplementary Table 2. Palaeohydrology data for Trou au Natron.
- Supplementary Table 3. Palaeohydrology data for the Era Kohor.

Supplementary References

Supplementary Notes

General geologic setting

The Tibesti Volcano Complex (TVC) in the central Sahara covers more than 100,000 km² and is among the world's major examples of intracontinental hot spot volcanoes. The main volcanic units in the TVC are plateau volcanism, central composite volcanoes and ignimbritic shield sheets [1]. Precambrian crystalline basement underlies the Tibesti massif but is also exposed and forms a core of intrusive and metamorphic rocks that are surrounded by Palaeozoic and younger sedimentary sequences[2-4]. These sedimentary sequences form deep basins that separate the Tibesti from other massifs to the west and east. To the south, the Tibesti massif is connected to Precambrian units that are overlain by post-Palaeozoic sequences[2,5]. Northward, sedimentary rocks of Palaeozoic, Mesozoic and Tertiary ages form a thickening wedge[2]. Volcanic activity began in the Oligocene[2] and intensified during the Quaternary. The ignimbritic, shield-like volcano Emi Koussi is the largest volcano of the TVC and was formed during an active sequence around 1.3 Ma[2,6], but volcanic eruptions occurred until the recent past, demonstrated by postvolcanic phenomena[7]. The 9 by 12 km large Emi Koussi summit caldera with the Era Kohor sub-crater (EKC; 4.14 km²) developed during the youngest of six volcanic phases of the TVC[2]. The western part of the TVC with the 3,296 m high strato-like volcano Pic Toussidè represents the youngest volcanic activity (less than 100 ky [3]). The older, neighbouring Yirrigué caldera is partly flooded by lava flows from Pic Toussidé and cut in the SW by the younger Trou au Natron crater (TaN; c. 40 km²), which also developed during the youngest volcanic phase. Noteworthy is the presence of well-defined rims and planar floors within the craters that do not point to asymmetrical or multicyclic subsidence [2].

Lithology

The base of section W566 (19°50.400’N / 18°33.101’E at 2774 m asl) consists of 10 cm (145-135 cm) of sand, gravel and small volcanic detritus material. This is followed by 60 cm (135-75 cm) of whitish, non-carbonaceous diatomite with a few gray to ochre sandy layers at 135-134 cm, 114-113 cm, and 88-86 cm and a few but consistently recorded Characeae stem fragments occur. Between 75 cm to 52 cm the monolith did not reach the laboratory intact and samples were taken at the base (75-70 cm) in the middle (65-62 cm) and at the top (52-57 cm). The upper 52 cm consist of whitish diatomite with few, ochre-coloured sand and gravel layers (46-42 cm and 22-21 cm), as well as rarely recorded Characeae stem fragments. Calcite contents increase within the upper 20 cm up to 6‰.

Section JK48 (20°58.349’N / 16°33.157’ E at 1560 m asl) bottoms out on sand, fine gravel and talus material, some 20 m above the crater floor c. 130 m west of the crater’s centre, and exposes 231 cm of lacustrine sediments, mainly limy to calcareous diatomite with varying sand contents. The entire section shows no signs of desiccation but is finely stratified with sub-mm thick layers of diatom debris and silty to fine-sandy particles, mainly quartz. The highest carbonate contents reach 32.2% and consist of calcite throughout this section. Other main components are – in decreasing contents – amorphous silica, quartz, feldspars and phyllosilicates. Charophyte stems and shales of snails occur only rarely in section JK48. Due to erosion and deflation at the top of JK48, no sediments of a final lake phase are exposed.

Section W99 (20°57.016’N / 16°33.110’E at 1890 m asl) on the southern slope of the TaN is situated 330 m above JK48 and bottoms out on sand, gravel and scree. The section exposes 329 cm of lacustrine sediments composed of carbonate-rich diatomite (up to 42.2% carbonates). Between the base (329 cm) and 113 cm, the carbonates consist solely of calcite but this changes towards the top to calcite and Aragonite (from 113 cm onwards), and to calcite, Aragonite and Mg-calcite (between 59 cm and the top). Similar to section JK48, other main components are – in decreasing contents – amorphous silica, quartz, feldspars and phyllosilicates. From 260 cm onward, mollusc shell fragments and Characeae stems occur increasingly towards the top of the yardang. Section W99 is also truncated by erosion at the top.

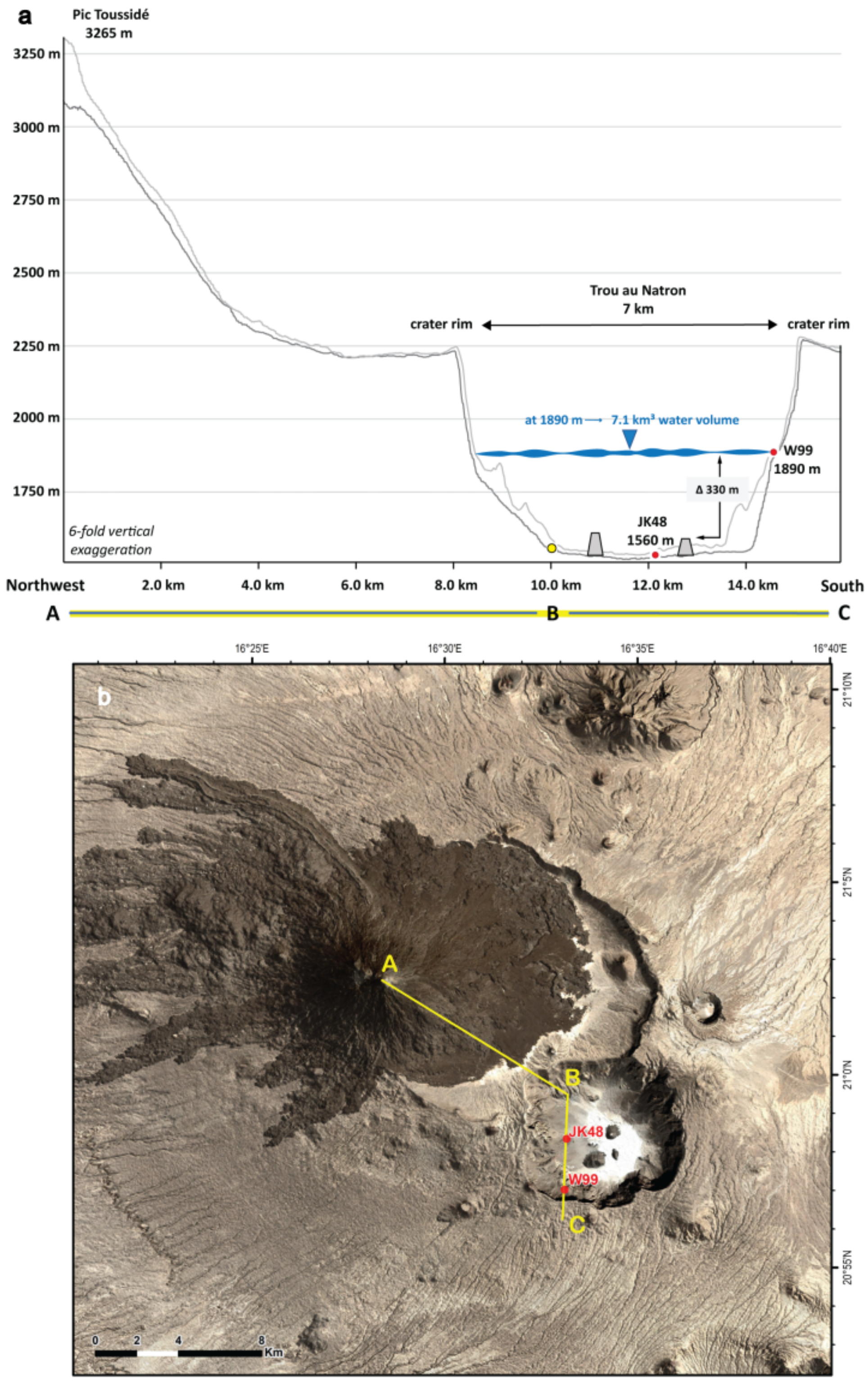

Supplementary Fig. 1 Schematic profile from the Pic Toussidé into the Trou au Natron. **a,** Topographic profile from the Pic Toussidé into the Trou au Natron crater with the deduced palaeolake depth and water volume; **b,** satellite image of the stratovolcano Pic Toussidé, the Yirrigué caldera and the Trou au Natron crater. Data sources: **a** JAXA ALOS DSM World 3D-30m (eorc.jaxa.jp), **b** ESA Copernicus Sentinel-2 (browser.dataspace.copernicus.eu), all publicly available.

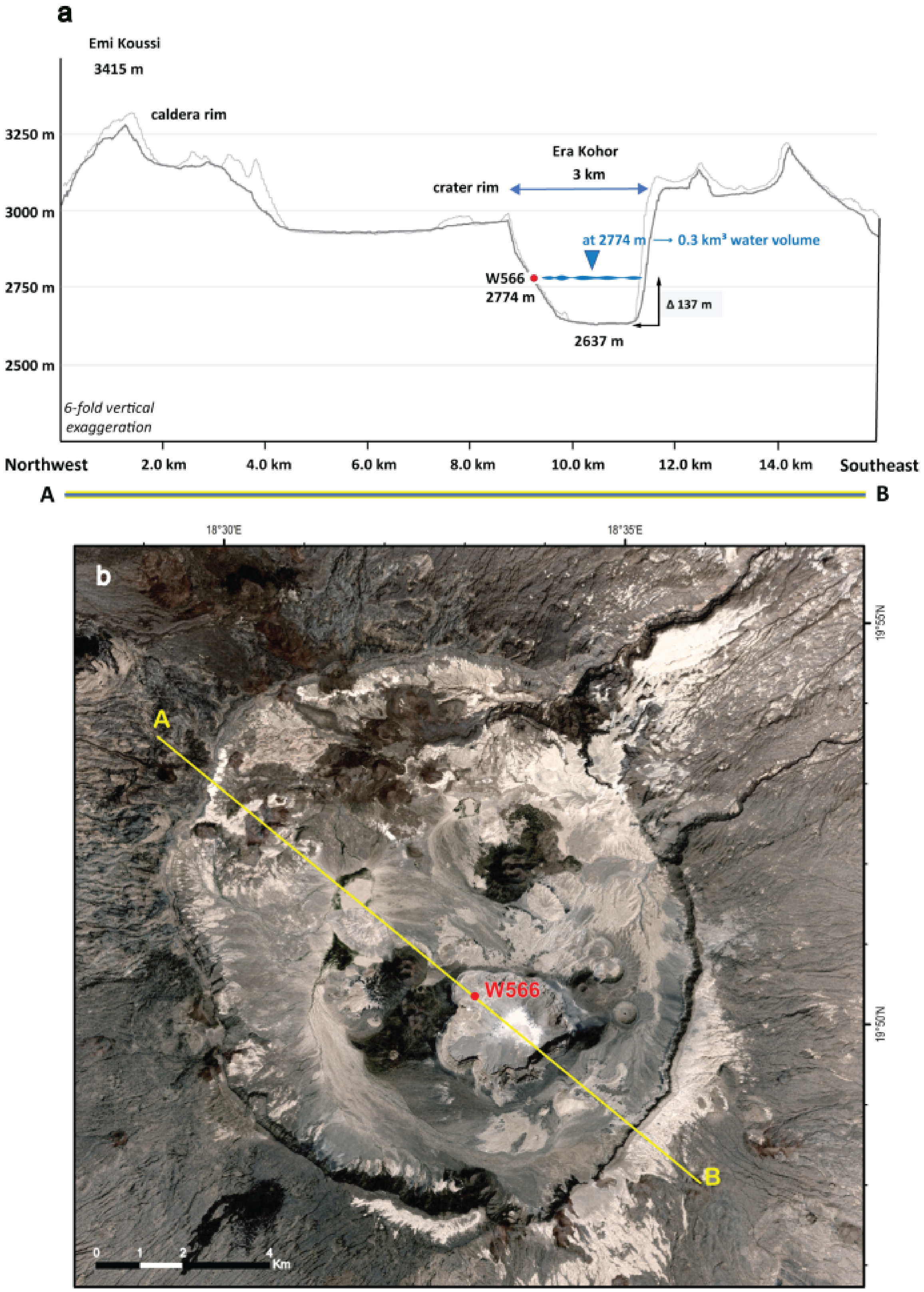

Supplementary Fig. 2 Schematic profile from the Emi Koussi summit caldera with the Era Kohor crater. **a**, Topographic profile from the peak of the Emi Koussi across the summit caldera through the secondary Era Kohor crater and the deduced palaeolake depth and volume. **b**, Satellite image of the Emi Koussi summit caldera. Data sources: **a**, JAXA ALOS DSM World 3D-30m (eorc.jaxa.jp), **b**, ESA Copernicus Sentinel-2 (browser.dataspace.copernicus.eu), all publicly available.

Dating and age depth model

A combination of different protocols[8-13] were applied to prepare the samples for radiocarbon dating. The preparation steps were as follows: (1) treatment with ~10% HCl (heated) for calcareous samples; (2) treatment with ~10% KOH (heated); (3) dense-media separation with sodium-polytungstate (SPT; ~2,1 g/cm^3); (4) microscopic analysis of the residues to decide on the next steps of the processing procedure; (5) density separation (1.6-1.2 g/cm^3) with CsCl or with 2.1 g/cm^3 SPT, with microscopic control of the potential different phases; (7) sieving (40, 10,6 µm depending on sample).

The age depth models of the two sections JK48 and W99 (Trou au Natron) document a Holocene deposition of limnic sediments between 9.8 and 4.8 cal ky BP. The base of the JK48 sequence, positioned near the bottom of the caldera, is dated to 9.8 cal ky BP and the top to 5.4 cal ky BP. The higher positioned W99 sequence is dated at the base to 7.8 cal ky BP and to 4.5 cal ky BP at the top.

However, for both sequences, erosion (e.g. deflation, slope wash) of the youngest sediments must be assumed, so that the top of the sections does not represent the termination of lacustrine sedimentation within the Trou au Natron.

The age depth model for W566 (Era Kohor crater within the summit caldera of the Emi Koussi) is discussed in Dinies et al.[11] and applied in Yacoub et al.[15], also indicates a Holocene formation of lake sediments between 9.65 and 5.5 cal kyr BP.

Supplementary Table 1

cm below top	Lab-number	Material dated	^{14}C age BP	calibrated a BP*	Reference
**Trou au Natron**					
W99 (0-329 cm)					
63-64	Beta-423374	*Tamarix* charcoal	4520±30	5310-5050	[19]Yacoub et al. 2023
106-107	Beta-423375	*Tamarix* charcoal	5090±30	5915-5750	[19]Yacoub et al. 2023
149-150	Poz-176179	cpp	6360±50	7422-7166	this paper
150-151	Poz-169929	cpp	6020±70	7155-6671	this paper
206-210	Poz-122316	cpp	6210±40	7250-6990	[19]Yacoub et al. 2023
275-276	Beta-453615	*Tamarix* charcoal	6330±30	7320-7170	[19]Yacoub et al. 2023
288-289	Poz-125904	cpp	6500±50	7560-7310	[19]Yacoub et al. 2023
290-297	Poz-122315	cpp	6080±80	7160-6750	[19]Yacoub et al. 2023
299-300	Poz-169927	cpp	6530±80	7572-7280	this paper
JK48 (0-231 cm)					
40-41	Beta - 453611	upp	5420±20	6290-6130	[19]Yacoub et al. 2023
92-93	Poz-176154	cpp	7550±50	8420-8200	this paper
128-129	Poz-126143	cpp	6860±130	7960-7490	[19]Yacoub et al. 2023
150-153	Poz-122363	cpp	7540±50	8420-8200	[19]Yacoub et al. 2023
195-198	Beta - 470620	cpp	7320±60	8320-8010	[19]Yacoub et al. 2023
221-222	Poz-122364	cpp	8640±40	9690-9540	[19]Yacoub et al. 2023
222-223	Poz-122317	cpp	8570±110	9910-9300	[19]Yacoub et al. 2023
**Era Kohor**					
W566 (0-147 cm)					
0-15	Beta - 441716	cpp	4720±30	5580-5320	[18]Dinies et al 2021
33-36	Poz-122314	cpp	7480±50	8380-8180	[18]Dinies et al 2021
51-52	Poz-176153	cpp	6430±90	7510-7170	this paper
76	Poz-176155	cpp	7700±50	8590-8400	this paper
95-96	Poz-122366	cpp	7700+/-40	8590-8400	[18]Dinies et al 2021
123-125.5	Beta - 441715	cpp	8360±40	9480-9160	[18]Dinies et al 2021
136-137	Poz-122367	cpp	7510+/-50	8400-8195	[18]Dinies et al 2021

Supplementary Table 1. Radiocarbon datings. The age depth models of the palaeolake sections are mainly based on published datings[14,15]. Additional datings were performed for several depths to assess the age depth models. These additional data are listed in Supplementary Table 1. All datings were calibrated with OxCal (https://c14.arch.ox.ac.uk/oxcal.html) using IntCal20[45]. Charred plant particles concentrate (cpp); uncharred plant parenchym (upp).

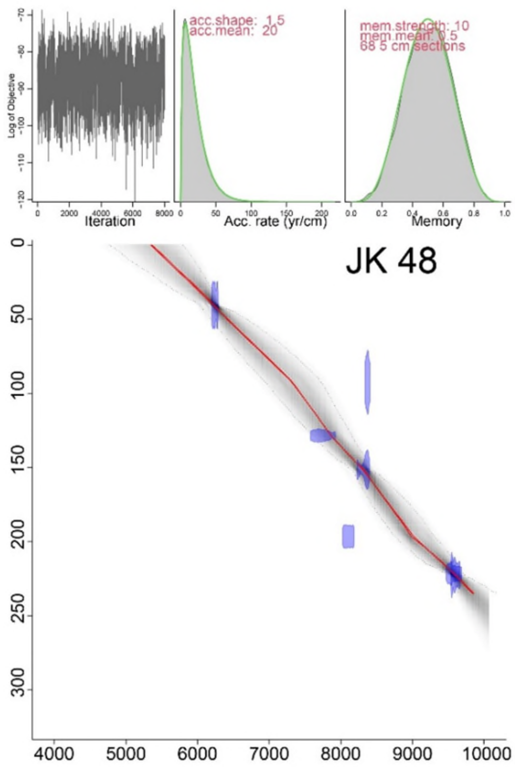

Supplementary Fig. 3. Age depth model of the Trou au Natron palaeolake section JK48. rBacon (3.0.0) was used to create the models (standard configuration), with mean ages for the respective depths plotted in red. Depth below section top are plotted in cm on the x-axis, ages as cal years BP on the y-axis.

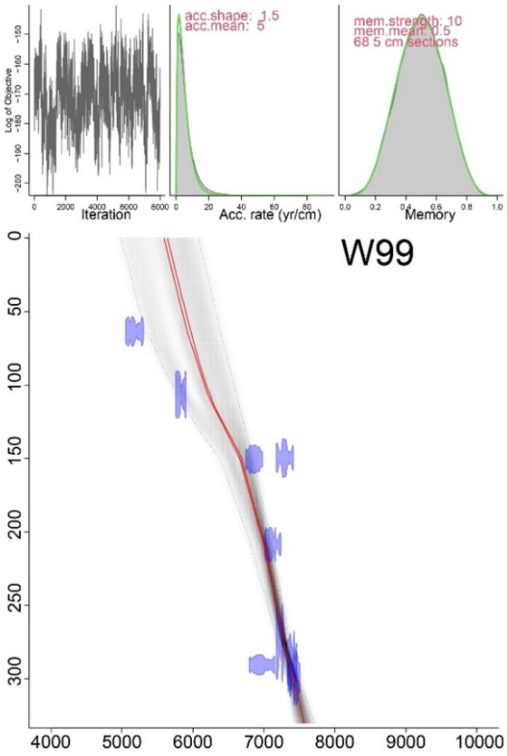

Supplementary Fig. 4. Age depth model of the Trou au Natron palaeolake section W99. rBacon (3.0.0) was used to create the models (standard configuration), with mean ages for the respective depths plotted in red. Depth below section top are plotted in cm on the x-axis, ages as cal years BP on the y-axis.

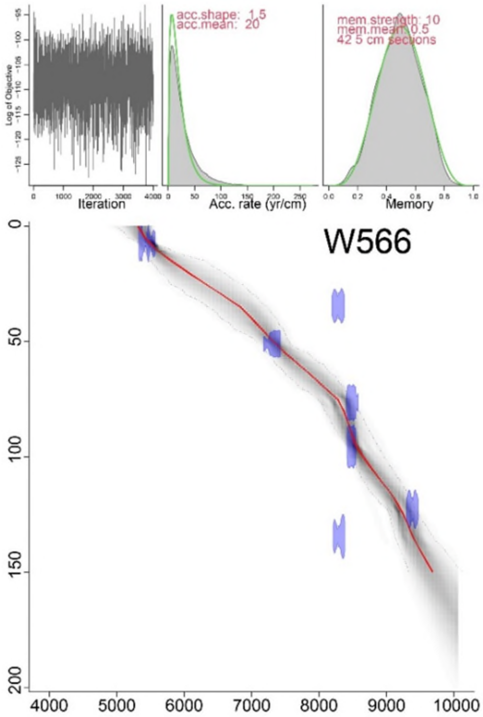

Supplementary Fig. 5. Age depth model of the Era Kohor palaeolake section W566. rBacon (3.0.0) was used to create the model (standard configuration), with mean ages for the respective depths plotted in red. Depth below section top are plotted in cm on the x-axis, ages as cal years BP on the y-axis.

Palaeolake evolution

Within the Trou au Natron crater, two lake sediment sequences were studied. Eight lake phases were differentiated, representing lake evolution in the TaN between 9.7 and 4.8 cal ky BP. The initial lake phase (Phase I: c. 9.7 – 9.3 cal ky BP) displays higher Mn/Fe ratios and increased authigenic calcite precipitation with $\delta^{18}O_{carb}$ values around -2.5‰ during lake formation and consequently lower lake levels. Thereafter, the lake stabilizes and deepens (II) until c. 8.2 cal ky BP, when again increased authigenic calcite precipitation with δ18O_{carb} values between -5 and -2.5‰ and higher Mn/Fe ratios reflect reductions in P/E. This short-term lake level lowering (III) is related to the 8.2 ky Northern Hemisphere cooling event[16]. During a following deep lake phase (IV: 7.9 to 7.3 cal ky BP), lake sediments with low $\delta^{18}O_{carb}$ values of -6‰ were also deposited at the position of W99, some 330 m above and 2000 years after the initial lake phase started near the crater floor. The deepest lake phase (V) lasts from 7.3 to 6.6 cal ky BP, before first signs of progressively drier conditions are recorded by increased authigenic calcite production in section JK48 and are more clearly expressed in section W99 with higher Mn/Fe ratios and authigenic calcite with increasing $\delta^{18}O_{carb}$ values (lake phase VI). This mid-Holocene drying trend continues. Increasing $\delta^{18}O_{carb}$ and $\delta^{13}C_{carb}$ values of up to +5‰ and +7‰, respectively, are indicative of decreasing P/E towards the end of the NAHHP. In comparison to the pro-fundal section JK48, strong authigenic carbonate production dominates throughout the littoral (W99). In addition to authigenic calcite (V-VIII), successively aragonite from 6.2 cal ky BP (lake phase VII) and magnesium-calcite from 5.4 cal ky BP onwards are formed, representing continuously increasing water salinities and a trend toward progressively drier conditions (lake phase VIII). In this part of section W99, $\delta^{18}O_{carb}$ values of around +4‰ indicate low but stable P/E ratios, while strongly fluctuating and decreasing $\delta^{13}C_{carb}$ values are interpreted to reflect oxidative decomposition of organic material during periods without permanent water coverage. Due to erosion, a final lake phase after 4.8 cal ky BP in the TaN is not represented in either section.

Section W566 in the Era Kohor crater is located 130 m above the present crater floor and represents a palaeolake between 9.5 and 5.4 cal ky BP[14,15]. The topographic position of W566 point to lake formation starting prior to 9.5 cal ky BP (Fig.2). Fluctuating (sagewort) *Artemisia* and Poaceae frequencies indicate the persistence of cold-steppe vegetation during the lake phase. An aridity index based on vegetation reconstruction points to wetter conditions up to 8.5 cal ky BP (Phase A; Fig.2: 9.5 – 8.5 cal ky BP), followed by a period of reduced humidity (B: 8.5 – 7.9 cal ky BP)[14]. Slightly moister conditions are documented by Mn/Fe ratios until 6.2 cal ky BP (C), parallel to documented increased humidity (lake phases V and VI) in the Trou au Natron. Authigenic calcite production started around 6.2 cal ky BP (D; corresponding to VII in the Trou au Natron), reflecting lower lake levels at least until 5.4 cal ky BP and the onset of the long-term drying trend at the Era Kohor with values for $\delta^{18}O_{carb}$ of around +4.5‰. The top of section W566 is truncated by erosion.

The low amount of weighted $\delta^{18}O_{ANN}$ of meteoric water [17] in the Tibesti mountains was influenced by the altitude and amount effect and can explain the lowest $\delta^{18}O_{carb}$ values of -6‰ around 7.2 cal ky BP in the deepest part of the W99 section, which are comparable assuming equilibrium oxygen isotope fractionation at around 17°C summer temperature. Enhanced productivity of algae results in increasing pH and – due to the preferred uptake of ^{12}C – in generally higher $\delta^{13}C_{carb}$ values. In closed lakes, CO_2 degassing under arid conditions leads to an enrichment of ^{18}O and ^{13}C in surface water and positive covariation in associated authigenic carbonates[18]. The continuously increasing $\delta^{18}O_{carb}$ and $\delta^{13}C_{carb}$ values in both profiles reflect decreasing precipitation and increasing evaporation due to progressive aridity.

Supplementary Tables 2 and 3

	P [mm/y]	*PE* [mm/y]	*R* [mm/y]	*G* [mm/y]	*PE/(P+R)*
Year 1	914 (387)	1136 (1247)	296 (125)	-73 (735)	0.94 (2.43)
Year 2	1589 (757)	1257 (1202)	514 (245)	-846 (200)	0.60 (1.20)
Year 3	1964 (962)	1182 (1230)	635 (311)	-1417 (-43)	0.45 (0.97)
Year 4	827 (340)	1147 (1243)	268 (110)	52 (794)	1.05 (2.77)
Year 5	1343 (622)	1292 (1189)	435 (201)	-486 (365)	0.73 (1.44)
Year 6	1093 (485)	1193 (1226)	354 (157)	-254 (584)	0.82 (1.91)
Year 7	1538 (729)	1274 (1196)	498 (361)	-762 (231)	0.63 (1.24)
Year 8	1144 (513)	1026 (1288)	370 (166)	-488 (609)	0.68 (1.90)
Year 9	1404 (655)	1085 (1266)	454 (212)	-773 (399)	0.58 (1.46)
Year 10	1887 (920)	1155 (1240)	611 (298)	-1343 (-22)	0.46 (1.02)
Year 11	1077 (477)	1155 (1240)	349 (154)	-271 (609)	0.81 (1.97)
Year 12	1814 (880)	1214 (1218)	587 (285)	-1186 (54)	0.51 (1.05)
Year 13	934 (398)	1136 (1247)	302 (129)	-100 (720)	0.92 (2.37)
Year 14	1119 (500)	1120 (1253)	362 (162)	-361 (592)	0.76 (1.89)
Year 15	1180 (533)	1209 (1220)	382 (173)	-353 (515)	0.77 (1.73)
Mean	1322 (610)	1172 (1234)	428 (198)	-577 (426)	0.71 (1.69)
Std	365 (200)	71 (26)	118 (65)	463 (275)	0.18 (0.56)
Std [%]	28 (33)	6 (2)	28 (33)	80 (65)	25 (33)

Supplementary Table 2. Palaeohydrology data for Trou au Natron. Annual precipitation *P*, potential evaporation *PE*, surface runoff *R*, groundwater flow *G*, and the ratio of potential evaporation to the sum of precipitation and runoff, indicating a measure of dryness. The upper values in each cell are the values taken from the 5 km simulation, the values in brackets refer to the results of the 40 km simulation. All Values are given as water column changes. Dark blue colour indicates the strong monsoon year, light blue, the weak monsoon year.

	P [mm/y]	*PE* [mm/y]	*R* [mm/y]	*G*[mm/y]	*PE/(P+R)*
Year 1	236<br>(164)	1153<br>(1278)	198<br>(137)	719<br>(977)	2.66<br>(4.25)
Year 2	478<br>(450)	870<br>(1225)	400<br>(377)	-7<br>(398)	0.99<br>(1.48)
Year 3	530<br>(512)	1105<br>(1269)	444<br>(429)	131<br>(327)	1.13<br>(1.35)
Year 4	227<br>(153)	1057<br>(1260)	190<br>(128)	640<br>(979)	2.53<br>(4.49)
Year 5	481<br>(455)	1105<br>(1269)	403<br>(381)	221<br>(434)	1.25<br>(1.52)
Year 6	290<br>(228)	1084<br>(1265)	243<br>(191)	551<br>(847)	2.03<br>(3.02)
Year 7	401<br>(359)	977<br>(1245)	336<br>(301)	241<br>(528)	1.33<br>(1.89)
Year 8	337<br>(284)	1084<br>(1265)	282<br>(238)	464<br>(744)	1.75<br>(2.43)
Year 9	585<br>(577)	1164<br>(1280)	489<br>(483)	90<br>(220)	1.08<br>(1.21)
Year 10	583<br>(576)	918<br>(1234)	488<br>(482)	-153<br>(177)	0.89<br>(1.17)
Year 11	346<br>(294)	1116<br>(1271)	289<br>(246)	481<br>(731)	1.76<br>(2.36)
Year 12	463<br>(433)	1068<br>(1262)	388<br>(363)	216<br>(466)	1.25<br>(1.58)
Year 13	357<br>(307)	945<br>(1239)	299<br>(257)	289<br>(675)	1.44<br>(2.20)
Year 14	423<br>(385)	1009<br>(1251)	354<br>(322)	233<br>(544)	1.30<br>(1.77)
Year 15	391<br>(348)	1025<br>(1254)	328<br>(291)	306<br>(615)	1.43<br>(1.96)
Mean	408<br>(368)	1045<br>(1258)	342<br>(308)	295<br>(581)	1.52<br>(2.18)
Std	113<br>(134)	86<br>(16)	94<br>(112)	240<br>(250)	0.53<br>(1.02)
Std [%]	28<br>(36)	8<br>(1)	28<br>(36)	81<br>(43)	35<br>(48)

Supplementary Table 3. Same as Supplementary Table 2, but for the palaeohydrology data for the Era Kohor.

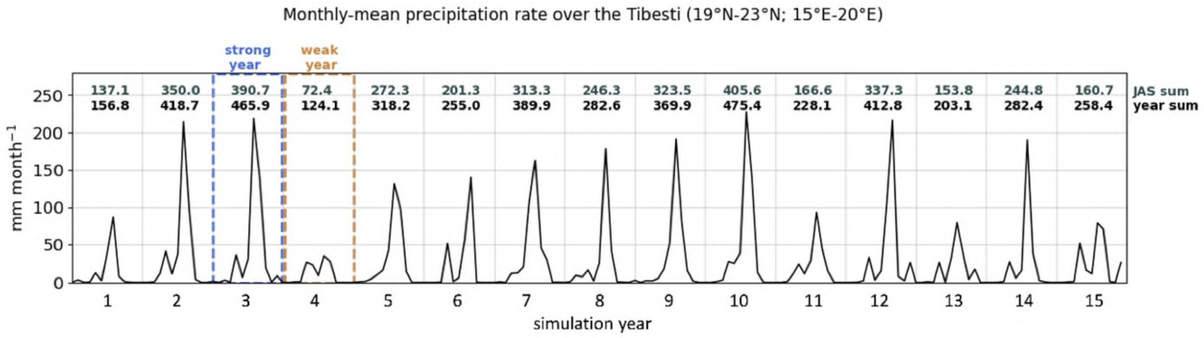

Supplementary Fig. 6. Timeseries of monthly mean precipitation rates averaged over the Tibesti Mountain in the 40 km simulation. The series displays the last 15 years of the 40 km-horizontal resolution spin-up simulation over the region of 19°-23°N/15°-20°E. The simulation year 1 is the year 6974 y BP in the astronomical calendar. The numbers at the top give the mean JAS (July-August-September) precipitation rate for each year and the annual precipitation sum, which on average amount to 252 +/- 101 mm and 309 +/- 109 mm, respectively. The blue and orange dashed boxes display the selected strong and weak monsoon years, respectively.

Assessment of the climate modelling results

Reconstructions of mid-Holocene precipitation for North Africa are only available as annual mean anomalies to present-day levels[19]. Due to the lack of a modern simulation, we cannot directly compare our simulated results with these reconstructions. Therefore, we perform a relative comparison of the rainfall distribution, assuming that the spatial pattern of monsoon precipitation determines the spatial pattern of the annual precipitation. To this end, we choose a reference site (grid point centre: 11°E, 13°N) in the reconstructions by Bartlein et al.[19] and the corresponding grid cell in the model. This location is chosen because out of the few proxy sites, this site and the corresponding grid cell are located roughly in the centre of the main monsoon rain belt in the high-resolultion (5 km grid size) simulation at around 7 ky BP and because the simulated precipitation sum over the grid box considered approximately equals the reconstructed precipitation sum. We calculate the rainfall difference between this reference and the reconstructions at all other sites and grid cells (Supplementary Fig. 7a, b). Choosing another reference site in the main monsoon rain belt would only alter the numbers, but not the qualitative results and conclusion.

The north-south rainfall gradient is stronger in the model than in the reconstructions. Over the Tibesti Mountains, the reconstructions show slightly lower precipitation rates than in the monsoon rain belt, whereas the model shows slightly higher precipitation rates. The model might slightly overestimate, but generally capture the orographic intensification of rainfall over the Tibesti Mountains. We argue that the reconstructions are based on pollen assemblages and probably aggregate the climate signal of several centuries. Our simulations provide only two snapshots. Given the strong variability of the West African monsoon system, these snapshot simulations and even the 15 years of simulations at 40 km-resolution may underestimate the variability in precipitation. It cannot be ruled out that years of stronger and weaker rainfall occurred.

The outstanding high rainfall rates in the Tibesti Mountains compared to the surrounding are also observed during present-day in years with strong monsoon activity (Supplementary Fig. 7e). In August 2018, for example, precipitation on the Tibesti reached sums as observed for the Southern Sahel region (TAMSAT data [20-22]). This underlines the importance of the mountains for triggering precipitation in the Sahara region. Therefore, the high precipitation rates over the Tibesti Mountains in our mid-Holocene simulations in which the rainfall in the monsoon rain belt is much higher than today are considered plausible.

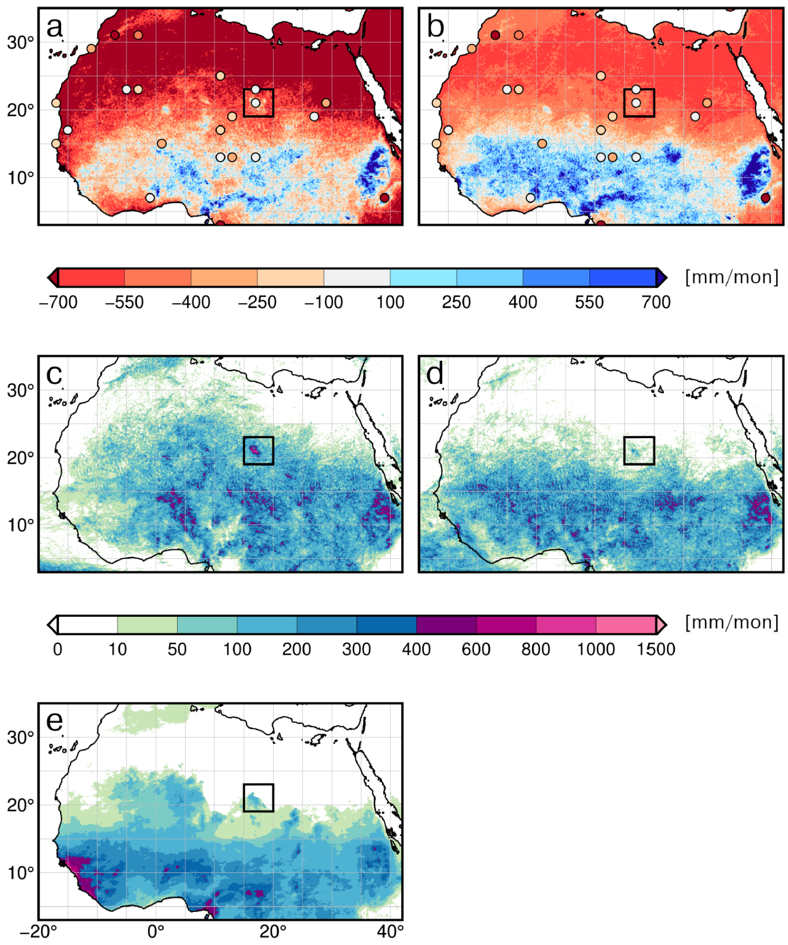

Supplementary Fig. 7. Evaluation of the climate model simulation with respect to precipitation. Since a modern simulation has not been performed and precipitation reconstructions are only available as anomalies to present-day climate, the simulation is evaluated based on the spatial relative precipitation pattern. We compare the simulated precipitation difference between each grid cell and a reference grid cell (centre: 11°E,13°N) in the main monsoon rain belt, for (**a**) the strong monsoon season and (**b**) the weak monsoon season, respectively, with corresponding precipitation gradients based on reconstructions[19]. In addition, the precipitation pattern during August is displayed for (**c**) the strong monsoon simulation, (**d**) the weak monsoon simulation, and (**e**) a strong monsoon year at present-day (i.e. year 2018CE, taken from TAMSAT satellite data[20-22]. Data sources: **a**) and **b**) the ICON-NWP simulations in 5km used in this study (shadings) and pollen-based precipitation reconstructions[19]; **c**) and **d**) ICON-NWP simulations in 5km used in this study; **e**) TAMSAT satellite data[20-22], https://www.tamsat.org.uk/data; Copyright University of Reading, 2017 last access: May 8, 2024). This dataset is made available under the terms of the Creative Commons Attribution 4.0 International license (CC BY 4.0).

Supplementary References

[1] Vincent P. M. The evolution of the Tibesti volcanic province, eastern Sahara. In *African magmatism and tectonics* (eds. Clifford, T.N. & Gass, I.G.) (Oliver and Boyd, Edinburgh, 1970).

[2] Permenter, J. L., & Oppenheimer, C. Volcanoes of the Tibesti massif (Chad, northern Africa). *Bulletin of Volcanology* **69**, 609-626 (2007).

[3] Ball P. W. et al. Quantifying asthenospheric and lithospheric controls on mafic magmatism across North Africa. *Geochemistry, Geophysics, Geosystems* **20**: 3520-3555. https://doi.org/10.1029/2019GC008303)

[4] Deniel, C., Vincent, P. M., Beauvilain, A., & Gourgaud, A. The Cenozoic volcanic province of Tibesti (Sahara of Chad): major units, chronology, and structural features. *Bulletin of Volcanology* **77**, 1-21 (2015).

[5] Ghuma, M. A., & Rogers, J. J. Geology, geochemistry, and tectonic setting of the Ben Ghnema batholith, Tibesti massif, southern Libya. *Geological Society of America Bulletin* **89(9)**, 1351-1358 (1978).

[6] Gourgaud, A., & Vincent, P. M. Petrology of two continental alkaline intraplate series at Emi Koussi volcano, Tibesti, Chad. *Journal of volcanology and geothermal research* ***129*****(4)**, 261-290 (2004).

[7] Reese, D., Okrusch, M. & Kaiser, K. Die Vulkanite des Trou au Natron im westlichen Tibesti Gebirge (Zentral Sahara). *Berl. Geogr. Abh.* **24**, 7-37 (1972).

[8] Brown, T. A., Farwell, G. W., Grootes, P. M., & Schmidt, F. H. Radiocarbon AMS dating of pollen extracted from peat samples. *Radiocarbon* **34(3)**, 550-556 (1992).

[9] Regnéll, J., & Everitt, E. Preparative centrifugation—a new method for preparing pollen concentrates suitable for radiocarbon dating by AMS. *Vegetation History and Archaeobotany* **5**, 201-205 (1996).

[10] Nakagawa, T., et al. Dense-media separation as a more efficient pollen extraction method for use with organic sediment/deposit samples: comparison with the conventional method. *Boreas* **27(1)**, 15-24 (1998).

[11] Vandergoes, M. J., & Prior, C. A. AMS dating of pollen concentrates—a methodological study of late Quaternary sediments from south Westland, New Zealand. *Radiocarbon* **45(3)**, 479-491 (2003).

[12] Chester, P. I., & Prior, C. A. An AMS 14C pollen-dated sediment and pollen sequence from the late Holocene, southern coastal Hawke's Bay, New Zealand. *Radiocarbon* **46(2)**, 721-731 (2004).

[13] Piotrowska, N., Bluszcz, A., Demske, D., Granoszewski, W., & Heumann, G. Extraction and AMS radiocarbon dating of pollen from Lake Baikal sediments. *Radiocarbon* **46(1)**, 181-187 (2004).

[14] Dinies, M., et al. Holocene high-altitude vegetation dynamics on Emi Koussi, Tibesti mountains (Chad, central Sahara). In *Quaternary Vegetation Dynamics—The African Pollen Database (ed. Runge, J.) Ch. 4 (CRC Press, Leiden, 2021).*

[15] Yacoub, A. N., et al. The African Holocene Humid Period in the Tibesti mountains (central Sahara, Chad): Climate reconstruction inferred from fossil diatoms and their oxygen isotope composition. *Quat. Sci. Rev.* **308** 108099 (2023).

[16] Alley, R. B., et al. Holocene climatic instability: A prominent, widespread event 8200 yr ago. *Geology* **25(6)**, 483-486 (1997).

[17] Terzer, S., Wassenaar, L. I., Araguás-Araguás, L. J., & Aggarwal, P. K. Global isoscapes for δ 18 O and δ 2 H in precipitation: improved prediction using regionalized climatic regression models. *Hydrology and Earth System Sciences* **17(11)**, 4713-4728 (2013).

[18]Horton, T. W., Defliese, W. F., Tripati, A. K., & Oze, C. Evaporation induced 18O and 13C enrichment in lake systems: A global perspective on hydrologic balance effects. *Quat. Sc. Rev.* **131**, 365-379 (2016).

[19]Bartlein, P. J., et al. Pollen-based continental climate reconstructions at 6 and 21 ka: a global synthesis. *Climate Dynamics* **37**, 775-802 (2011).

[20]Maidment, R. I., Allan, R. P., & Black, E. Recent observed and simulated changes in precipitation over Africa. *Geophysical Research Letters* **42(19)**, 8155-8164 (2015).

[21]Tarnavsky, E., Grimes, D., Maidment, R., Black, E., Allan, R., Stringer, M., Chadwick, R. & F. Kayitakire (2014). Extension of the TAMSAT Satellite-based Rainfall Monitoring over Africa and from 1983 to present. *Journal of Applied Meteorology and Climatology* **53 (12)**: 2805-2822. DOI: 10.1175/JAMC-D-14-0016.1

[22]Maidment, R., D. Grimes, R. P. Allan, E. Tarnavsky, M. Stringer, T. Hewison, R. Roebeling & E. Black (2014). The 30 year TAMSAT African Rainfall Climatology And Time series (TARCAT) data set. *Journal of Geophysical Research* **119**: 10,619-10,644. DOI: 10.1002/2014JD021927.
